# An Employment Intervention Program (Work2Prevent) for Young Men Who Have Sex With Men and Transgender Youth of Color (Phase 2): Protocol for a Single-Arm Mixed Methods Pilot Test to Assess Feasibility and Acceptability

**DOI:** 10.2196/16401

**Published:** 2020-08-10

**Authors:** Brandon J Hill, Darnell N Motley, Kris Rosentel, Alicia VandeVusse, Robert Garofalo, John A Schneider, Lisa M Kuhns, Michele D Kipke, Sari Reisner, Betty M Rupp, Maria Sanchez, Micah McCumber, Laura Renshaw, Rachel West Goolsby, Matthew Shane Loop

**Affiliations:** 1 Planned Parenthood Great Plains Overland Park, KS United States; 2 Center for Interdisciplinary Inquiry and Innovation in Sexual and Reproductive Health Department of Obstetrics and Gynecology University of Chicago Chicago, IL United States; 3 Guttmacher Institute New York City, NY United States; 4 Division of Adolescent Medicine, Ann & Robert H Lurie Children’s Hospital Department of Pediatrics, Feinberg School of Medicine Northwestern University Chicago, IL United States; 5 Department of Medicine University of Chicago Chicago, IL United States; 6 Division of Research on Children, Youth, and Families Children's Hospital Los Angeles Los Angeles, CA United States; 7 Fenway Health The Fenway Institute Boston, MA United States; 8 Collaborative Studies Coordinating Center Department of Biostatistics, Gillings School of Global Public Health University of North Carolina at Chapel Hill Chapel Hill, NC United States

**Keywords:** HIV/AIDS, YMSM, YTW, GNC youth, LGBTQ, unemployment, homelessness, sex work

## Abstract

**Background:**

Young cisgender men who have sex with men (YMSM), young transgender women (YTW), and gender nonconforming (GNC) youth of color face substantial economic and health disparities. In particular, HIV risk and infection among these groups remains a significant public health issue. In 2017, 17% of all new HIV diagnoses were attributed to male-to-male sexual contact among adolescents and young adults aged 13 to 24 years. However, such disparities cannot be attributed to individual-level factors alone but rather are situated within larger social and structural contexts that marginalize and predispose YMSM, YTW, and GNC youth of color to increased HIV exposure. Addressing social and structural risk factors requires intervention on distal drivers of HIV risk, including employment and economic stability. The Work2Prevent (W2P) study aims to target economic stability through job readiness and employment as a structural-level intervention for preventing adolescent and young adult HIV among black and Latinx YMSM, YTW, and GNC youth. This study seeks to assess intervention feasibility and acceptability in the target populations and determine preliminary efficacy of the intervention to increase employment and reduce sexual risk behaviors.

**Objective:**

The goal of the research is to pilot-test a tailored, theoretically informed employment intervention program among YMSM, YTW, and GNC youth of color. This intervention was adapted from Increased Individual Income and Independence, an existing evidence-based employment program for HIV-positive adults during phase 1 of the W2P study.

**Methods:**

The employment intervention will be pilot-tested among vulnerable YMSM, YTW, and GNC youth of color in a single-arm pre-post trial to assess feasibility, acceptability, and preliminary estimates of efficacy.

**Results:**

Research activities began in March 2018 and were completed in November 2019. Overall, 5 participants were enrolled in the pretest and 51 participants were enrolled in the pilot.

**Conclusions:**

Interventions that address the social and structural drivers of HIV exposure and infection are sorely needed in order to successfully bend the curve in the adolescent and young adult HIV epidemic. Employment as prevention has the potential to be a scalable intervention that can be deployed among this group.

**Trial Registration:**

ClinicalTrials.gov NCT03313310; https://clinicaltrials.gov/ct2/show/NCT03313310

**International Registered Report Identifier (IRRID):**

DERR1-10.2196/16401

## Introduction

### Background

Youth assigned male at birth who have male sexual partners, including young cisgender men who have sex with men (YMSM), young transgender women (YTW), and gender nonconforming (GNC) youth, face substantial economic and health disparities. In particular, HIV risk and infection among YMSM, YTW, and GNC youth remains a significant public health problem. In the United States, YMSM, YTW, and GNC youth experience high rates of HIV infection [1,2]. In 2017, 17% of all new HIV diagnoses were attributed to male-to-male sexual contact among adolescents and young adults aged 13 to 24 years [1]. Additionally, 75% of adolescent and young adult HIV diagnoses were among black and Latinx individuals [1]. Epidemiological HIV estimates for transgender populations are limited due to a lack of existing data. However, a meta-analysis of US studies involving trans women found an average HIV prevalence rate of 14% across studies that included laboratory testing [2]. In this meta-analysis, prevalence rates were higher among black trans women at 44% [2].

However, these disparities cannot be understood solely in the context of individual-level risk behavior given that there are multiple social and structural factors that increase risk for HIV exposure and acquisition among YMSM, YTW, and GNC youth of color [3-10]. Despite advancements in lesbian, gay, bisexual, transgender, and queer (LGBTQ) rights, LGBTQ people face persistent stigma, discrimination, and victimization in school, the workplace, housing, and health care [11-14]. Such inequities are met with limited legal protections as few state laws specifically protect LBGTQ people. The consequences of this discrimination and lack of protections may be particularly pronounced for LGBTQ people of color, who face intersectional forms of discrimination and structural marginalization; a high proportion of YMSM, YTW, and GNC youth of color live in poverty; experience high rates of homelessness, unemployment, and violence; and have limited access to HIV and other health and human services [14-20].

Furthermore, these experiences of social and economic marginalization contribute to increased risk for HIV exposure and infection through their impacts on social determinants of health (eg, availability of pre-exposure prophylaxis [PrEP] providers in the community) as well as coping and survival behaviors (eg, substance use, sex work) [14-16,18,20]. In particular, financial insecurity and socioeconomic disconnection may increase engagement with survival sex work or sex in exchange for money, drugs, food, and housing among YMSM, YTW, and GNC youth of color [21-23]. Engagement with survival sex work can place these individuals at heightened risk for HIV and sexully transmitted diseases (STIs) by increasing exposure to higher prevalence sexual networks, increasing their number of sexual partners, and presenting challenges to negotiating condom use [21-23].

Structural-level interventions have the potential to increase agency in members of marginalized groups and can facilitate health-positive actions that benefit the individual and the community [24]. Often focused on distal drivers of poor health, structural-level interventions can promote uptake of health-positive behaviors and improve access to health-promotive environments [24,25]. Given the ways that economic instability may place YMSM, YTW, and GNC youth of color at higher risk for HIV acquisition, structural intervention to promote economic stability may serve to allow these youth to enact health-promoting behaviors. Accordingly, employment as prevention has the potential to be a scalable intervention that can be deployed among this group. Phase 1 of this study has already been published [26].

### Rationale for Employment as HIV Prevention

Faced with few economic options and protections, YMSM, YTW, and GNC youth of color may migrate to nontraditional economies or unregulated work as a means of survival. In a study conducted by Adolescent Medicine Trials Network for HIV/AIDS Interventions (ATN) members in Los Angeles and Chicago, 76% of 151 YTW aged 15 to 24 years reported engaging in sex work, with 35% in the past 3 months [5]. Among HIV-positive YTW of color living in Washington, DC, 23% were involved in sex work—underscoring the link between adolescent and young adult sex work and HIV exposure [27]. In a large US study of YMSM (N=3316, median age 19 years), roughly 12% reported engaging in sex work in the past 6 months [28]. Sex work and HIV risk are further complicated by drug and alcohol abuse [16]. In order to effectively target economic stability as a route toward reducing HIV risk, there is an acute need for scalable, low-cost but potentially high impact structural-level interventions that address the distal drivers of economic marginalization and adolescent and young adult HIV infection [24,25,29,30]. The objective of the Work2Prevent (W2P) study is to adapt and pilot-test Increased Individual Income and Independence (iFOUR), an effective, theoretically driven employment program for HIV-positive adults [31-34] to the needs of vulnerable YMSM, YTW, and GNC youth of color aged 16 to 24 years.

### Theoretical Framework

The iFOUR intervention draws on the theoretical framework of the health belief model (HBM) [35], a widely used expectancy value model of health behavior change, and the conceptual framework of supported employment (SE), a model in which individuals with physical or intellectual disabilities or impairments, mental health issues, or chronic conditions are assisted with identifying their own capabilities and obtaining employment [36,37]. The objective of the iFOUR intervention is to help HIV-positive individuals identify barriers to obtaining employment, increase the perceived benefits of employment, and assess perceptions of the severity of their illness in order to increase behavioral intentions and self-efficacy for employment. Further, iFOUR participants gain the tools and skills needed to effectively seek, secure, and maintain employment and increase economic independence and stability [31-34].

In order to adapt and tailor the iFOUR intervention to the needs of adolescents and young adults of color, the intervention draws on positive youth development (PYD) in which young people understand, value, and develop external and internal assets such as community support, empowerment to act, clear boundaries, constructive use of time, commitment to learning, positive self-concept, and social and emotional competency [38-40]. PYD approaches orient young people toward future goals, develop the skills necessary to engage youth in real-world roles and activities, and build or fortify young peoples’ relationships with social networks [40]. PYD builds from resiliency research in assuming that all youth are capable of achieving positive health outcomes despite challenges they may face in their environment [38]. The adapted intervention will draw on PYD to provide the support, relationship-building skills, and increased social and emotional competency shown to help youth succeed in employment.

## Methods

### Conceptual Model

**Figure 1:**
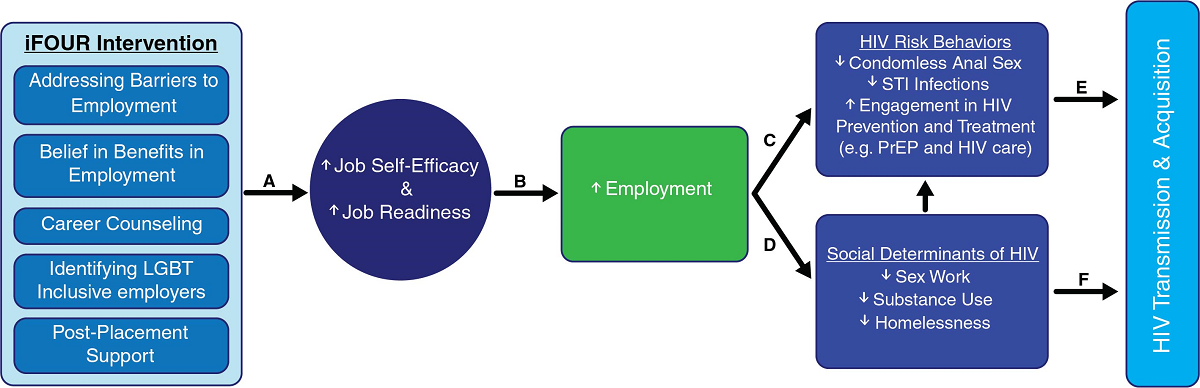
Conceptual model.

The W2P conceptual model shown in Figure 1 draws on the existing iFOUR theoretical framework to hypothesize the potential relationship between adolescent and young adult employment and HIV risk. The W2P model proposes that employment and subsequent economic connection and stability serve as a structural-level intervention for HIV prevention among adolescents and young adults. The hypothesis is that the adapted and tailored iFOUR intervention will facilitate increased job self-efficacy and job readiness (path A) and ultimately increase employment placement and maintenance (path B). Further, establishing economic stability will decrease engagement in HIV risk behaviors, increase HIV prevention and care (path C), and decrease involvement with known social determinants of HIV such as sex work and substance use (path D), which are directly linked to HIV transmission and acquisition among YMSM, YTW, and GNC youth of color (paths E and F).

### Study Design

W2P uses a mixed-methods design. Phase 1 involves the adaptation of relevant intervention components from the existing evidence-based iFOUR employment program for HIV-positive adults [31-34] to YMSM, YTW, and GNC youth of color. Phase 2, the topic of this paper, consists of pretesting the intervention and study assessments and then running a single-arm pilot test of the adapted intervention to assess feasibility and acceptability with YMSM, YTW, and GNC youth of color, as well as provide preliminary estimates of efficacy using pre-post comparisons.

### Ethics, Consent, and Institutional Board Approval

W2P has been reviewed and approved by the University of Chicago institutional review board (IRB# 16-1152). Informed consent for this study is obtained in person by study staff before any study-related activities take place.

### Participants and Study Setting

Study participants include up to 75 black or African American and Hispanic or Latinx YMSM, YTW, and GNC youth. Inclusion criteria include (1) being assigned male at birth, (2) reporting ever having sex with men, (3) identifying as African American or black or Hispanic or Latinx, (4) aged 16 to 24 years, (5) self-report HIV negative or unknown status, (6) English-speaking, (7) currently unemployed but seeking employment or employed only part-time, defined as working 35 hours or less on average per week, and (8) able to attend a 4-session workshop. All study visits are conducted at the University of Chicago Center for Interdisciplinary Inquiry and Innovation in Sexual and Reproductive Health (Ci3).

### Recruitment

Planned participant recruitment efforts include the distribution and posting of printed materials such as flyers, hand bills, and branded merchandise; online postings on websites and social media such as Facebook and Twitter; advertisements such as Chicago Transit Authority posters; and chats through the mobile app Jack’d. Study staff will also actively recruit from primary clinics serving YMSM, YTW, and GNC youth such as Howard Brown Health during their youth drop-in programs and at local gathering places and events frequented by the target population such as night clubs, LGBTQ centers, House & Ball events, Black Pride events, Pride Fest, and community outreach HIV testing events. Interested participants complete a prescreen survey to assess eligibility. All interested participants are contacted and informed whether they are eligible. Eligible and interested participants are scheduled for baseline study visits.

### Visit Schedule and Data Collection

W2P consists of data collection across 3 time points, at baseline, postintervention, and 8-month postintervention, as referenced in Table 1. A 4-session intervention workshop series occurs between the first and second time points.

**Table 1:** Study procedures.

Study procedures	Baseline	Intervention	Postintervention	Month 8 follow-up
HIV/STI^a^ testing	x			x
Substance use screening	x			x
ACASI survey^b^	x		x	x
Workshop sessions (4)		x		

^a^STI: sexually transmitted disease.

^b^ACASI: audio computer-assisted self-interview.

### Incentives

Study participants are offered compensation for their time. Pretest participants may receive up to US $260 total, while pilot-test participants may receive up to US $330 total for complete participation. Participants will receive US $30 for each study visit, completed at baseline, postintervention, and 8-month follow-up, up to US $40 for biological specimens at baseline and 8-month follow-up, if provided, and US $40 for each workshop session attended. Payment is provided in the form of cash or Visa gift card equivalent.

#### Pretest

Up to 5 participants will be recruited for a pretest of the baseline study visit, the 4-session workshop series, and the postintervention study visit. Pretest participants will not complete the follow-up assessment. The purpose of the pretest is to give study staff an opportunity to familiarize themselves with the study visit procedures and workshop curriculum. The pretest will also allow staff to determine if any final adjustments to procedures, study instruments, or the curriculum are needed prior to full rollout of the pilot testing. Participants who enroll in the pretest are not eligible to participate in the phase 2 pilot.

#### Baseline

Participants will complete informed consent, confirm eligibility, and then complete an audio computer-assisted self-interview (ACASI) survey using an iPad. Survey items include questions pertaining to demographics, sexual behaviors, HIV-risk behaviors, relationships, employment, income, substance use, and other structural variables such as homelessness, food insecurity, and health care use. Optional biologic samples will be collected from participants who consent to them. These samples include a finger stick for rapid HIV testing using the Determine HIV-1/2 Ag/Ab Combo (Abbott); a urine sample for drug screening, chlamydia, and gonorrhea testing; and anal and oral swabs for chlamydia and gonorrhea testing.

#### Intervention

Participants complete a 4-session intervention workshop adapted from the existing iFOUR program [31-33]. Session 1 focuses on goal setting and identifying strengths; session 2 on communication, networking, and job searching; session 3 on balancing work with health and wellness; and session 4 on preparing job application materials and interview preparation. This adaptation of the curriculum was informed by interviews and focus groups with the target population as well as feedback from a youth advisory board. The protocol for conducting interviews and focus groups is published elsewhere [26].

Workshops sessions are delivered by two facilitators in groups of 6 to 12 participants across the course of 2 weeks with 2 sessions per week. The W2P Career Readiness Workbook is used as a guide for all workshop sessions and is given to all study participants at the first session. Facilitators use an annotated W2P Facilitator Guide that provides detailed instruction on delivery of the intervention curriculum. During each session, facilitators complete a fidelity assessment to help ensure fidelity to the W2P Career Readiness Workbook and after each session complete a workshop debriefing form to capture any workshop notes or comments.

#### Postintervention

Once participants complete the workshop sessions, participants complete a postintervention ACASI survey using an iPad. Survey items include questions on workshop evaluation, job-seeking self-efficacy, and PrEP and HIV testing use.

#### Month 8 Follow-Up

The final study visit occurs 8 months after the intervention has been completed. During this visit, participants complete the baseline ACASI survey using an iPad and provide repeat biologic samples if they consented to them.

### Outcomes

#### Primary Outcomes

##### Information Systems Success Model Score

The Information Systems Success Model (ISSM) will be used to assess for intervention acceptability and satisfaction. The 21-item scale yields a total score and measures 4 subdomains: information quality, handbook quality, perceived usefulness, and overall satisfaction. This scale has been adapted from Horvath et al [41].

##### Workshop Completion

Workshop completion will be used to assess intervention feasibility. Workshop or intervention completion is defined as having attended at least 2 of the 4 workshop sessions and is measured by tracking participant attendance.

##### Change in Job-Seeking Self-Efficacy Scale Score

Job-seeking self-efficacy is defined as one’s perceived ability and confidence to perform job search and application activities. The 12-item Job-Seeking Self-Efficacy scale by Barlow et al [42] yields a total score where higher values indicate higher self-efficacy. Job-seeking self-efficacy has been found to be associated with employment in a previous study of transgender women of color [29].

##### Change in Protean Career Attitudes Scale Score

Protean career attitudes (PCAs) are defined as having self-direction in the pursuit of success in one’s work. PCAs have previously been found to be associated with positive career satisfaction and self-perceived success [43]. The validated 7-item scale by Porter et al [44] yields a total score and measures 2 subdomains: self-directed attitudes and values-driven attitudes.

#### Secondary Outcomes

##### Change in Self-Reported Hours Worked per Week

Hours worked per week is self-reported at baseline and at the 8-month follow-up visit. Change in hours worked per week from the baseline to the 8-month follow-up will be used to assess change in employment status.

##### Change in Self-Reported Sexual Risk Behaviors

Sexual risk behaviors are defined as self-reported engagement in the following behaviors during the previous 6 months [45]:

- Condomless anal intercourse (receptive or insertive) with cisgender male partner of unknown HIV status
- Anal intercourse (receptive or insertive) with 3 or more cisgender males
- Sex with cisgender male partner with an STI
- Condomless anal intercourse (receptive or insertive) with HIV+ cisgender male partner
- Anal intercourse (receptive or insertive) with condom failure
- Transactional sex work involvement

The previous 6 months refers to the 6 months prior to the baseline visit for the first assessment and the 6 months prior to the 8-month follow-up visit for the second assessment. Change in sexual risk behaviors will be defined as the change in self-reported behaviors from baseline to the 8-month follow-up.

##### Change in Chlamydia Test Result

Prevalence of chlamydia infections will be assessed at baseline and 8-month follow-up using oral, anal, and urine samples. Each of the 3 tests yields a positive or negative result. Change in chlamydia test result will be defined as the change from baseline to the 8-month follow-up. Oral, anal, and urine tests are treated as separate outcomes.

##### Change in Gonorrhea Test Result

Prevalence of gonorrhea infections will be assessed at baseline and 8-month follow-up using oral, anal, and urine samples. Each of the 3 tests yields a positive or negative result. Change in gonorrhea test result will be defined as the change from baseline to the 8-month follow-up. Oral, anal, and urine tests are treated as separate outcomes.

##### Reactive HIV Test

Testing for reactive or nonreactive HIV will be assessed at baseline and 8-month follow-up. The reactive HIV test outcome uses the 8-month follow-up result.

### Power

Given the exploratory nature of this study and limited access to this population, the analyses are not designed to have a specified level of statistical power. A repeated measures pre and post design is used to reduce the variability in the estimate of the treatment effect.

### Statistical Analysis

Descriptive statistics will be used to analyze the proportions and central tendencies for participant sociodemographic characteristics collected in the surveys. We will first generate frequencies, means, and other measures of central tendency as appropriate to describe our sample and outcomes at each of the 3 time points: baseline, postintervention, and 8-month follow-up.

All participants who are enrolled at baseline and complete the baseline ACASI will be included in the primary and secondary analyses as applicable. Analysis population participants will be included in all primary and secondary analyses for which their data for the specified outcome are not missing. Participants who do not attend any workshop sessions will not be included in analyses involving workshop evaluation. Primary analyses will assess intervention acceptability, satisfaction, and feasibility as well as change in job-seeking self-efficacy and PCA score. Secondary analyses will evaluate the intervention by comparing employment and sexual risk behaviors pre- and postintervention.

Changes in primary and secondary outcomes between baseline and 8-month follow-up will be assessed using paired *t* tests for continuous variables (eg, ISSM, job-seeking self-efficacy, and PCA scores) and the McNemar test for matched categorical variables (eg, STI results). We will use standard diagnostic tools to assess the appropriateness of the normality assumption and, if approximate normality of the residuals is not tenable, a nonparametric test for continuous paired data (ie, Wilcoxon sign-rank test) will be used. All hypothesis testing will be performed at an alpha level of 0.10, given the exploratory nature of the study. To the extent that data allows, multivariable analyses will adjust for sociodemographic characteristics, workshop attendance, baseline employment status, and study completeness. Analytical models will include linear regression or generalized linear models for continuous outcomes and logistic regression for binary outcomes.

Analysis of the primary and secondary outcomes are described in detail within the statistical analysis plan, which will be accessible on ClinicalTrials.gov once study results have been entered.

## Results

Phase 2 W2P research activities began in March 2018 and were completed in November 2019. Overall, 5 participants were enrolled in the pretest, and 51 participants were enrolled in the pilot.

## Discussion

The goal of this project is to pilot-test W2P, a structural-level employment intervention for YMSM, YTW, and GNC youth of color. Interventions that address the social and structural drivers of HIV exposure and infection are sorely needed in order to successfully bend the curve in the adolescent and young adult HIV epidemic. Although important for HIV prevention, few individual interventions consider the complex ecological factors that make YMSM, YTW, and GNC youth of color vulnerable to HIV. Thus, engagement with individual-level interventions, such as PrEP adherence and consistent condom use, may be impeded by broader issues such as homelessness, unemployment, and survival sex work. Addressing these factors is an important first step in mitigating risk for adolescent and young adult HIV.

Although employment is an important target for increasing economic stability and decreasing reliance on nontraditional economies such as survival sex work, one limitation of this protocol may be that an immediate individual reduction of HIV exposure may not be detectable. Often structural-level interventions rely on measurement of predictive outcomes that ultimately have downstream effects on health outcomes. To address this challenge, the study focuses on measures of job readiness, job-seeking self-efficacy, and career readiness as the strongest predictors of employment engagement and employability. Follow-up occurs at 8 months postintervention allowing participants time to enact skills and behaviors gained from the employment intervention. Additionally, there is a potential limitation that the results of the intervention may not be generalizable beyond urban YMSM, YTW, and GNC youth of color, as both the formative phase and intervention tailoring and refinement focused on the needs of this population. Subsequent adaptation and refinement may be necessary to engage youth outside of this target population.

If W2P demonstrates feasibility and acceptability among YMSM, YTW, and GNC youth of color in this pilot study, we plan to test the efficacy of the intervention in a multicity longitudinal trial across the ATN study sites. If W2P demonstrates efficacy, this intervention will provide vulnerable youth a tailored youth-focused way to gain employment and life-based skills necessary to achieve economic stability and ultimately reduce the propensity for HIV exposure and infection.

## Appendix

Multimedia Appendix 1 Peer review report with comments.

## Abbreviations

ACASI: audio computer-assisted self-interview
ATN: Adolescent Medicine Trials Network for HIV/AIDS Interventions
Ci3: Center for Interdisciplinary Inquiry and Innovation in Sexual and Reproductive Health
GNC: gender nonconforming
HBM: health belief model
iFOUR: Increased Individual Income and Independence
ISSM: Information Systems Success Model
LGBTQ: lesbian, gay, bisexual, transgender, queer
PCA: protean career attitudes
PrEP: pre-exposure prophylaxis
PYD: positive youth development
SE: supported employment
STI: sexually transmitted disease
W2P: Work2Prevent study
YMSM: young men who have sex with men
YTW: young transgender women
