# Supplementary material for: An Employment Intervention Program (Work2Prevent) for Young Men Who Have Sex With Men and Transgender Youth of Color (Phase 2): Protocol for a Single-Arm Mixed Methods Pilot Test to Assess Feasibility and Acceptability
Source: JMIR Res Protoc. 2020 Aug 10;9(8):e16401. doi: 10.2196/16401 (PMC7445605; doi:10.2196/16401)
Supplement: Multimedia Appendix 1 [file resprot_v9i8e16401_app1.docx]

**Reviewer U1: Overall Comments**
I suggest the authors review and clarify the gender and sex partner gender of the persons they describe in the paper. Specifically, I assume that “young men who have sex with men” refers only to cisgender men since the study is only for people assigned male sex at birth. If so, I would add “cisgender” to the description of this group, as transmen who have sex with men would not be included. I also think references to “male-to-male” sexual contact need to be clearer if it is cisgender or transgender men that are being discussed.

RESPONSE U1: We have added cisgender to the first place where we define YMSM. The reference to “male-to-male sexual contact” pulls the exact language used by the CDC in the referenced report. This is one of the transmission route categories that they use.

**Reviewer U2: Overall Comments**
Similarly, it was not clear to me if the transgender women and gender non-conforming youth had to have sex with men to be included, which I imagine they do. So, I would maybe describe the population being discussed as “cisgender men, transgender women, and gender non-conforming youth of color who have sex with men.” Another consideration is to just use “assigned male at birth” (AMAB) as you do in a couple places, so “persons of color who are assigned male at birth (AMAB) and have sex with men.”

RESPONSE U2: The very first sentence specifies that we are referring to individuals who are assigned male at birth and have sex with men for all gender categories. We then proceed with the abbreviations we have defined moving forward for ease of reading.

**Reviewer U3: Overall Comments**
I would also change “substance abuse” to “substance use” throughout.

RESPONSE U3: This revision has been made.

**Reviewer U4: Abstract Comments**
I am not familiar with including references in an abstract, especially if the citations they refer to are in the bibliography of the main paper, and would suggest removing from the abstract. It also made it a little confusing in the first paragraph of the Introduction when the second reference was to #10.

RESPONSE U4: These citations have been removed.

**Reviewer U5: Abstract Comments**
I felt that the abstract introduction was a little long, and could be shortened.

RESPONSE U5: This has been addressed.

**Reviewer U6: Abstract Comments**
In the abstract results I would include more information (will also discuss this below). At a minimum I would include the sample size in the abstract. Since it is now early November, the reference to follow-up continuing through the end of November may need to be changed.

RESPONSE U6: Thank you for your comment, this has been addressed.

**Reviewer U7: Abstract Comments**
I think the first sentence of the abstract conclusions should be the last sentence of the abstract introduction. In the abstract conclusions, I would not include what may be the results of a future multi-site trial, but focus on the conclusion of the present study.

RESPONSE U7: This has been addressed.

**Reviewer U8: Abstract Comments**
I would spell out the acronyms used in key words and maybe add “youth.”

RESPONSE U8: This has been addressed.

**Reviewer U9: Introduction Comments**
The first reference is to a webpage of multiple CDC reports. I think it would be helpful to cite the specific report that was the basis for this data.

RESPONSE U9: The correct url is now referenced.

**Reviewer U10: Introduction Comments**
I believe the 28% HIV prevalence referred to in the first paragraph is among transgender women only (i.e., not including transgender men), if so I would specify this since you refer to “transgender populations” in the preceding sentence which includes both transgender men and women.

RESPONSE U10: This has been addressed.

**Reviewer U11: Introduction Comments**
I thought the sentence describing reference #27 was awkward and would re-phrase, maybe “In a large US study of YMSM (N=3,316, median age = 19 years), roughly 12% reported engaging in sex work in the past 6 months.”

RESPONSE U11: This has been addressed.

**Reviewer U12: Methods Comments**
I think it may make more sense to start the Methods section with the conceptual model, followed by the study design.

RESPONSE U12: This has been addressed.

**Reviewer U13: Methods Comments**
I think the number of study participants who have been recruited (N=75) should be moved to the Results section.

RESPONSE U13: Clarified to show that the goal N was 75, and did not reflect the number recruited.

**Reviewer U14: Methods Comments**
I think the section re: inclusion criteria definitely needs to be reviewed for gender specifics and inclusion of sex partner gender. For example “being male” includes transmen, who I do not think are eligible, so I would remove this and just leave “assigned male at birth”. Further, “identifying as YMSM, YTW, or GNC youth” does not specify sex partner gender and I assume that a YTW or GNC youth who have sex only with cisgender women would not be eligible? I would clarify here who is eligible.

RESPONSE U14: We have updated the criteria based on your suggestions.

**Reviewer U15: Methods Comments**
I would move the “Visit Schedule & Data Collection” up to come before “Incentives," to provide a little more overall information about the study visits schedule before the details about incentives. Also, a figure or table of the study procedures and intervention would be useful in this section.

RESPONSE U15: This section has been moved as suggested and a table of study procedures is not included.

**Reviewer U16: Methods Comments**

In “Participants & Study Setting” would change “all study visits will be” to “all study visits are.”

RESPONSE U16: This has been addressed.

**Reviewer U17: Methods Comments**
In “Statistical Analysis” I think the paragraph about descriptive statistics of participant demographics should come first. I would clarify what is meant by “all participants who are enrolled at baseline will be included in the primary and secondary analyses” since all of your primary and secondary outcomes are changes over time or workshop completion – meaning people need to attend a workshop/complete follow-up surveys for outcome data. How would the people who enroll and don’t complete workshops or provide follow-up data be included?

RESPONSE U17: This section has been moved as suggested and we have updated text to better define our analysis populations.

**Reviewer U18: Methods Comments**

I think the authors should check the tests they propose for variables with more than 2 categories since McNemar’s is for 2x2 data and logistic regression is for binary variables.

RESPONSE U18: The MCNemar's will only be used for STI results, this has been clarified.

**Reviewer U19: Methods Comments**

It may be worth considering assessing baseline employment status as an interaction term if you think that the magnitude in change of some outcomes (e.g., job seeking self efficacy or career attitude) may be different for individuals who were not employed at baseline vs those working up to 35 hours per week.

RESPONSE U19: Thank you for your comment. We will include this interaction term in exploratory analyses. If data permit, given sufficient cell counts, we will consider including the interaction term in the main analysis.

**Reviewer U20: Results Comments**
I would have liked to see more results here. At least the sample size, and since everyone has enrolled maybe a baseline table? It would be informative and interesting.

RESPONSE U20: We have added the sample size and stated that data collection is complete. We plan to include a baseline table in our primary manuscript.

**Reviewer X1: Major comments**
It's unclear why the inclusion criteria required only self-report of HIV negative or unknown status if the goal was to test the intervention with youth with high risk for HIV infection - especially since HIV testing is offered as part of the study. It's also not clear if people who test HIV-positive at baseline will be excluded. Since HIV testing is optional, it's possible for an HIV positive person could self-report as HIV-negative, decline HIV testing, and participate in the pre-test and/or pilot test.

RESPONSE X1: The University of Chicago IRB stated that we could not make HIV-testing or any of the other biomedical tests a requirement to participate in workshops about employment because there was no medical justification. For this reason, we based the eligibility criteria off of self-report.

**Reviewer X2: Minor comments**

The first paragraph of the introduction cites data that is more than a decade old. Please cite the most recent data from Becasen et al. 2018 in AJPH.

RESPONSE X2: This has been addressed, we are now using a Becasen, et al. 2019 reference.

**Reviewer X3: Minor comments**
LGBTQ should be spelled out on first use.

RESPONSE X3: This has been addressed.

**Reviewer X4: Minor comments**

Third paragraph on Introduction: Social determinants of health are usually defined as factors such as where one lives, works, and plays. Access to PrEP does not fit within that definition. If the intention is to say the SDH impact access to PrEP, the sentence needs to be reworded.

RESPONSE X4: This has been addressed.

**Reviewer X5: Minor comments**

I recommend replacing "at-risk" with "vulnerable"

RESPONSE X5: This has been addressed.

**Reviewer X6: Minor comments**
Please clarify if the 5 individuals who take part in the pre-test will also be eligible for participation in the pilot test.

RESPONSE X6: This has been addressed.

**Reviewer X7: Minor comments**

The Intervention section refers the reader to a paper that is in draft. Since this paper is not available to the reader, it would be more useful to provide the necessary information in a supplemental appendix.

RESPONSE X7: The Phase 1 protocol paper is under review and we anticipate that it will be available in the same issue of JMIR as this paper if accepted. We have added it as a reference stating that it was submitted to JMIR. During copyediting if it has been accepted we will update the citation.

**Reviewer X8: Minor comments**

The secondary outcomes include references to anal sex with male partners. It would be helpful to know if this refers only to receptive anal sex or any anal sex. It would also be helpful to know if "male" refers to anyone with a penis (inclusive of some trans women and exclusive of some trans men) or to anyone who identifies as male or to anyone assigned male at birth. Please clarify.

RESPONSE X8: This has been addressed.

**Reviewer X9: Minor comments**
The change in the chlamydia and change in gonorrhea outcome is unclear. Does it refer to a change in prevalence in the aggregate or changes within individuals. It seems any changes in individuals over time would be difficult to interpret given the short duration of follow-up and the probably difficulty accessing data on treatment. For example, one person may test positive at baseline, never get re-exposed and never get treated and still be positive at 8 months. Another person may test positive at baseline, get treated and re-infected multiple times and be re-treated so that they test negative at 8 months. The second person has had risker behavior but their trajectory from positive to negative could erroneously be interpreted as reduced risk.

RESPONSE X9: It refers to change in prevalence given the short duration of follow-up and difficulty accessing data on treatment. We have clarified this.
